# Dismantle and rebuild: the importance of preparedness and self-efficacy before, during and after allogeneic haematopoietic cell transplantation

**DOI:** 10.1007/s11764-024-01622-2

**Published:** 2024-06-03

**Authors:** Katarina Holmberg, Karin Bergkvist, Yvonne Wengström, Carina Lundh Hagelin

**Affiliations:** 1 Karolinska Institutet, Stockholm, Sweden; 2 Sophiahemmet University, Stockholm, Sweden; 3 Karolinska University Hospital, Stockholm, Sweden; 4 Marie Cederschiöld University, Stockholm, Sweden

**Keywords:** Preparedness, Self-efficacy, Allogeneic haematopoietic cell transplantation, Haematology, Education, Person-centred care

## Abstract

**Purpose:**

The aim of this study was to explore patients’ experiences of being prepared for allogenic haematopoietic cell transplantation and to explore their perceived self-efficacy and preparedness for self-care after allogenic haematopoietic cell transplantation.

**Methods:**

Nine participants, who recently underwent allo-HCT, were interviewed regarding their views on preparedness, self-efficacy and self-care. The interviews were analysed using inductive qualitative content analysis.

**Results:**

An overarching theme, *Life is taken apart, then you have to know how to put the pieces together*, and four sub-themes: *Convert information into something understandable*; *Taking responsibility, maintaining and preparing for an uncertain time in life*; *Balancing vigilance with independence*; and *Reorientating in an altered body places new demands on self-care* illustrate the dismantlement of life during treatment and how actions and approaches can build a new life.

**Conclusions:**

Both participants and healthcare professionals prioritised preparing for allo-HCT in the period before admission. However, during admission, preparation decreased and the time was not used for preparatory learning. This meant that participants were well prepared for the acute phase but unprepared for life after completion of treatment. Among the participants, self-efficacy was good. They sought information about taking care of their health before and in the aftermath of allo-HCT.

**Implications for Cancer Survivors:**

This study provides insight into, and knowledge about, how patients prepare before, during and after treatment. This knowledge should primarily be directed towards healthcare professionals to be used for future patients who may need advice and support, as well as continued preparation for a life after transplantation.

## Introduction

Healthcare professionals (HCPs) are responsible for preparing patients for an examination or extensive treatment. Preparation is considered to be an important part of increasing a patient’s control in a vulnerable situation, with benefits such as improved recovery, less stress and an increased ability for self-management after cancer treatment [1, 2]. An unprepared patient risks the opposite, with poorer management of cancer symptoms [3].

Allogeneic haematopoietic cell transplantation (allo-HCT) aims to cure various haematological malignancies [4]. In order to predict the risks of undergoing allo-HCT, the patient’s condition is assessed regarding functional status, comorbidities and disease stage [5, 6]. The patient receives thorough information about the procedure and the risks regarding morbidity and mortality [7, 8]. The transplant procedure starts with chemotherapy as a pretreatment followed by transfusion of stem cells from an unrelated or related donor [9]. The effects of the treatment lead to the patient becoming susceptible to infections and requiring care in isolation for about a month. Side effects, such as indigestion, nausea, diarrhoea and mucositis, are also common [9, 10]. When both neutrophils and the patient’s condition start to recover, the isolation ceases and the patient is sent home with follow-up via outpatient care. At this point, it becomes the patient’s responsibility to independently monitor for side effects, signs of infections and adverse response from the immune system graft versus host disease (GvHD) [11]. They will manage their health status and medication with support from HCPs [10].

On completion of cancer treatment, the patient needs to monitor any physical and existential challenges that can occur that the patient needs to monitor, deal with and learn to cope with life after illness and treatment [12–15]. We have earlier reported that patients with a high symptom burden at 4 months after allo-HCT were more often on full-time sick leave and reported poorer general health 1 year after treatment [16]. Most frequently reported occurring symptoms were tiredness, physical weakness and disinterest in sex together with susceptibility to infection and feeling worried reported the first year post allo-HCT. Distressing symptoms varied more the first year after allo-HCT with difficulties eating, discomfort during sex, physical weakness together with loss of appetite and fragile genital membranes [16]. Sick leave in Sweden is covered by social insurance where all individuals with an income, unemployment benefits, studies and parenteral leave are covered for 1 year [17].

Self-efficacy is a person’s belief in their ability to put certain actions into practice to achieve a set goal [18] and is an important part of monitoring and managing self-care after allo-HCT [19]. Self-efficacy can be both taught and improved to impact patients’ behaviour and self-management; it is a significant part of the educational role of nursing [20, 21]. There are few studies that have examined self-efficacy [19] and patients’ experiences of the preparation during cancer treatment [22, 23]. The aim of this study was, therefore, to explore patients’ experiences of being prepared for allo-HCT and to explore perceived self-efficacy and preparedness for self-care after allo-HCT.

## Methods

### Study design

A qualitative descriptive design using individual interviews was adopted to gain a composite picture of the participants’ perceptions of the phenomena of preparedness and self-efficacy in allo-HCT [24].

### Setting and study participants

The study was conducted at a transplant centre in Sweden dedicated exclusively to allo-HCT. The inclusion criteria were adults ≥ 18 years who had undergone an allo-HCT during the past 12 months and who understood and spoke Swedish. The exclusion criteria were persons with cognitive impairments or in need of support regarding preparation and self-care, i.e. unable to take care of their own health without help from others such as nursing home residents, carers or personal assistants. The recruitment of participants was conducted on a voluntary basis with minimal consultation of medical records. Participants were sought via information displayed at the transplant centre’s outpatient clinic. Printed study information, clarifying the purpose of the study, was available in the waiting room. Contact details for the study coordinators were given so participants could get in touch and communicate their interest in participating. The first author planned the date, time and place of the interviews in consultation with the participants. Informed consent was included in the written study information and was signed and obtained from the participants before each interview.

Nine participants chose to participate described in Table 1. The interviews were performed between February 2022 and February 2023 and were held face-to-face or digitally according to the participant’s preference [25]. Four took place at the hospital outpatient clinic, three were conducted digitally, and two were held at other locations (the university and the participant’s workplace). Each interview was audio-recorded; total recorded time was 10 h, mean 75 min (min 44–max 135 min). Information power [26] was used as a guideline according to sample size.

**Table 1:** Characteristics of participants

Gender	Male	2
	Female	7
Age	Median (min–max)	70 (41–74)
Marital status	Married	5
	Cohabitant	1
	Single	3
Children	Yes	8
	No	1
Diagnosis	MDS	3
	AML	4
	Other	2
*Sick leave	Yes	2
	No	4
	*Partial	3
Months post allo-HCT	Median (min–max)	6 (3–12)

*Sick leave in Sweden is covered by social insurance where all individuals with an income, unemployment benefits, studies and parenteral leave are covered for 1 year. *Partial sick leave the level of compensation can be full time or part time at 25, 50 or 75% of regular working hours

### Data collection

A semi-structured interview guide was formulated by the research group based on the objective of the study. All authors are RNs and have extensive experience in haematology, oncology and allo-HCT. The interview guide was pre-tested by patient members of the Swedish Blood Cancer Association for usability and comprehensibility. It consisted of open-ended questions and suggestions for follow-up questions, as described in Table 2.

**Table 2:** Questions from the interview guide

Timeline	Question	Suggestions for follow-up questions
Before admission	- Can you tell me about who prepared you for the hospitalisation and the transplant and what they did?	- What helped you? - Was anything lacking?
	- Can you tell me about your own preparation before admission?	- Has any activity been significant for you and your preparation before admission?
During admission	- Can you tell me about your experience of how you were prepared when you were hospitalised?	- Who prepared you? - When during the care period? - In what way?
	- Can you tell me about how you prepared yourself during the period of hospitalisation?	- Was any activity important to you in preparing for your time on the ward?
Before discharge	- Can you tell me about the preparations for discharge and afterwards?	- Who prepared you for discharge? - When? - In what way?
	- Can you tell me about your preparations for discharge from the ward?	- Has any activity been significant for you and your preparation before being discharged? - In what way did you felt prepared when you were discharged?
Prepared for self-care	- Can you tell me if you felt prepared for the self-care that you perform?	- In what way?
(Self-efficacy) Confidence in own ability to perform self-care	- Can you tell me about your confidence (Self-efficacy) in your own ability to self-care?	- When do you feel confident in your ability, when is it difficult?

### Analysis

The data were analysed using inductive qualitative content analysis [27]. Each interview was transcribed verbatim in close performance of the interview to get a sense of the content as a whole. Transcription was done by the first author and contributed to further repeated reading of the text. The questions from the interview guide were used as a template to structure the text in a timeline: pre-admission, during admission and before discharge, since the conversation during the interview went back and forth in time as the participants recalled their experiences.

In this analysis, interview text, meaning units, condensed meaning units with interpretation of underlying meaning, sub-themes and themes were used [27]. Excerpts from the interviews were categorised and units of meaning related to each other were colour coded and condensed, preserving the core of what was expressed. In the condensed units, connections and patterns were sought and discussed back and forth in dialogue by the first and fourth authors. Patterns that emerged were summarised with a brief description and then merged with other similar descriptions. Descriptions of the phenomena appearing in the text consisted of abstractions and interpretations made by the research group. These were revised and merged, and similarities and differences between participants’ experiences were brought together and further discussed. Four subcategories constitute the latent content of the analysis and shape the overarching theme [27] (see Table 3). The results are presented with summaries and quotes in the results section [24].

**Table 3:** Examples of text, meaning units, condensed meaning units, sub-themes and theme

Text	Meaning unit	Condensed meaning unit	Sub-theme	Theme
…they told me a lot of things at this meeting before. He (the physician) talked for almost an hour non-stop, yes, but it was too much… you have to let it sink in and it will settle Participant no 2	He talked for almost an hour non-stop, it was too much at the time but settled down after a while	Conditions for transforming given information into preparedness	Convert information into something understandable	Life is taken apart, then you have to know how to put the pieces together
I exercise a lot and thought that I should be in the best possible shape, so… what should I say, I can do this, I’m strong Participant no 1	I exercise a lot and thought I will be in the best possible strong shape	Physical preparedness	Taking responsibility, maintaining and preparing for an uncertain time in life	
Yes, I think you try to do the best you can, then there may be questions. And now they still come home twice a week (home care). So that’s great. It is such a security that you can ask questions about whether you have done something right or wrong Participant no 9	You do the best you can, they still come to my home twice a week and it gives me such security as you can ask questions	The ability to ask if I am doing right or wrong	Balancing vigilance with independence	
I had to be prepared for it to happen, but then when it does happen, it’s obvious that it’s tough because of the eyes and mouth, and I felt that I couldn’t cope with much more. I thought that it was very hard when it happened… Participant no 4	Had to be prepared for side effects, but then when it happened it was hard. Despite preparations, there was the feeling of not being able to manage side effects	Despite preparations, there was the feeling of not being able to manage side effects	Reorientating in an altered body places new demands on self-care	

As the data were collected at one centre in Sweden and the number of participants is small, descriptive data are presented at group rather than individual level. Quotes are anonymised by referring to participants 1, 2, etc., to maintain confidentiality [28]. Counselling could be offered if the interview evoked feelings that needed to be handled professionally. This study followed the criteria for reporting qualitative research (COREQ) [29] and was performed in line with the principles of the Declaration of Helsinki [30]. Approval was granted by the Swedish Ethical Review Authority (Dnr, 202003996) and (Dnr, 2021–03865).

## Results

**Fig. 1:**
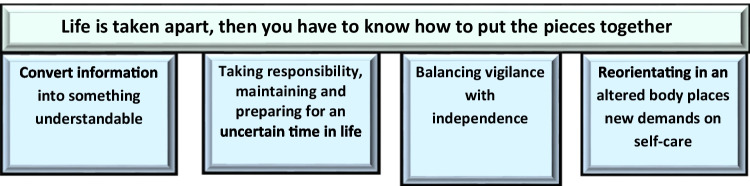
Overarching theme and sub-themes

The overarching theme, *Life is taken apart, then you have to know how to put the pieces together*, developed from the four sub-themes outlined in Fig. 1. The overall theme captures the dismantlement of life that can happen while undergoing allo-HCT, and how actions and approaches (presented in the four sub-themes) can rebuild life again.

### Convert information into something understandable

The information conveyed by the HCPs at the pre-meeting in the clinic was perceived by the participants as preparatory for the transplant process. It consisted of standardised information about guidelines, routines and statistics for allo-HCT. Written information was obtained describing the entire transplant procedure and late complications. Giving guidance more tailored to each individual was the coordinator’s responsibility. The coordinators were registered nurses (RNs) and their assignments ended at the patient’s enrolment. The coordinators were described as being very important in the preparations for admission. Participants reported that preparatory information was mostly provided before admission but also occurred during ward rounds and informal conversations, mainly with physicians and nurse assistants. RNs were rarely mentioned in the participants’ narratives, being referred to as friendly but busy.

Accessibility to HCPs and the timing of the conversations were key components in perceiving encounters as rewarding and being able to transform the information into something manageable. Other valuable components were when participants felt seen and listened to during a dialogue. Receiving encouragement and confirmation that everything had gone according to plan instilled hope and confidence, both in the care and in themselves.

“*She (physiotherapist) came in and was really great. She really cheered me up. My strength wasn’t much worse when I left compared to when I arrived. It was very positive and a bit therapeutic for me….*” Participant no 1.

Avoidant responses and too much information about the risks associated with the treatment contributed to doubt and mistrust and fed existential thoughts. Participants reported that information was often repeated and excessive, especially at enrolment and on discharge. According to the participants’ statements, an overabundance of information made its transformation into preparation more difficult in terms of understanding, perception and assimilation. Discharge was described as sudden and overwhelming with little or no opportunity to prepare or be prepared in advance.

“*…that day was incredibly intense. After a few weeks where not much happened. I mean, there was a lot with the healthcare in the early stage, but then, in these latter weeks, when you waited to get ready for going home, it was calmer until the day of discharge, when suddenly everyone would talk to me and I was so tired! I was so tired when I went home so yes, it was awful!*” Participant no 4.

### Taking responsibility, maintaining and preparing for an uncertain time in life

Participants’ own preparations for allo-HCT were physical, mental, cognitive, social, existential, emotional and practical. Most were carried out before admission. During treatment and before discharge, it was difficult to prepare as the information from HCPs was mainly medical. The participants wanted to be in good shape and took the opportunity to practise their favourite sports as physical preparation before admission. The focus on physical activity was more or less consistent for all participants. There was an awareness that it was important for rehabilitation, but during treatment and isolation it became difficult to be as active as desired.

Grasping the intangible sense of what an allo-HCT entails was described as difficult, despite repeated information from HCPs. Understanding all the aspects of the transplant process was something that required time to prepare for, both mentally and cognitively.

“*It would have been good to know how to think after six months. What do you have to think about? There was a lot of good information before the transplant, the brochure I got home was very detailed. But no such information has been received afterwards*.” Participant no 5.

The participants described needing support from HCPs, relatives and friends. Social preparations could include arranging for live-in help after discharge or organising children’s day-care from a contagion perspective. Existential preparation could consist of ensuring that relatives had access to bank accounts, codes and passwords or writing a will, in case something serious were to happen. Telling the children was something addressed in existential emotional preparedness, encompassing an awareness of life and its finitude.

“*I arranged a day with each child and we did what they wanted. I didn’t say I was going to do this risky thing, just that I was going to be in hospital for a long time. I wanted some time alone with each of them. At the back of my mind were thoughts that things could go wrong.*” Participant no 3.

Practical preparedness involved searching for information preadmission, often via the internet or by joining patient associations, through social media, or contacting people who had undergone an allo-HCT. Aiming for an alternative perspective to those provided by the healthcare system was sought. Practical preparations also included keeping busy with activities, such as reading, needlework and doing puzzles. Some described it being difficult to occupy themselves and ended up in a state of limbo where they mostly watched TV or stared at the ceiling.

### Balancing vigilance with independence

The sub-theme balancing vigilance with independence relates to the participants’ confidence in their ability to manage self-care after allo-HCT. The participants had to deal with making decisions in everyday life after transplantation. Several struggled with the question of when the restrictions following the allo-HCT would end.

“*The tricky thing is what is a risk. How should I relate to things that are risky for me. In the beginning it was easier because then it’s like: You have to be careful of everything. Then you have to somehow start trying to live a little more and it has been up to me to sort it out.*” Participant no 8.

For a long time, many of the participants continued to live a limited life in terms of socialising, handling pets, doing housework and avoiding certain foods from a better to be safe than sorry perspective. The advice given was described as inconsistent due to a lack of continuity at return visits and not seeing the same physician twice. This could lead to prolonged restrictions in life, brooding or a mild rebellion against the guidelines. Participants described the need for evaluating risk vs benefit when making choices. *Is it worth exposing myself to the risk of getting sick or should I opt out and stay home* was the question they needed to confront before journeys, dinners or meeting grandchildren and others.

“*I’ve probably tried to live a little more than they said you should but I’ve been careful. I’ve sat in a cafe and eaten cake, to get some fat and they don’t think you should go into a cafe … So I’ve pushed their boundaries a bit compared to the recommendations I was given, but I’ve done it with some kind of caution, but for me it’s been important to be able to live.*” Participant no 3.

### Reorientating in an altered body places new demands on self-care

Despite a lack of preparation prior to discharge, all participants described feeling ready and willing to go home. They also expressed confidence in taking care of their own health at home. A gradual return to everyday life was described when energy levels were low and their body felt unfamiliar.

“*… You feel a bit like a ticking bomb when you keep checking to see if something has happened to your skin or if something has happened here or there? You never really know... how long afterwards can something happen? Could something urgent happen or could there be some side effects or something else? You know something can happen. But I’m not afraid of it.*” Participant no 9.

The participants’ words revealed that they were unprepared for what type of rehabilitation might be relevant for them. They were also unprepared for how weight and muscle loss affected rehabilitation, nutritional problems, ever-present anxiety or sleep difficulties months after allo-HCT; they experienced intrusive thoughts dreams of death, funerals and memories of the past. Suffering from late complications was perceived as a serious setback despite the information given. Self-care had to be developed progressively due to a weaker body with side effects from treatment and graft versus host disease. The main focus during return visits to the physician was on the medical part with little or no time for existential questions or information.

“*If a doctor had told me, I would have been mentally prepared for these thoughts after a few months, and it’s not unique to me, we know that, but there are many who experience the same thing, so you should be better prepared for what is to come.*” Participant no 1.

## Discussion

In general, participants reported being well prepared prior to allo-HCT but less prepared for the consequences of treatment. This corresponds with results from previous studies where participants felt unprepared for the next step after completion of cancer treatment [3, 31–33]. Communication, coordination, education, participation of patients and collaboration in the care team are elements that should be included for a successful discharge [34]. The discharge procedure in this study was uniformly described as disorganised, with little opportunity to prepare or participate. In this study, a factor to consider is the participants’ median age of 70 years. This might require a carefully planned discharge, taking into account age-related limitations in communication [35]. The overload of information was perceived as repetitive and tiring, and at that point participants admitted that they just wanted to go home. However, wanting to go home is not the same as being prepared for going home [36]; low preparedness pre-discharge can be linked to low coping at home [37]. This was evident when participants expressed being ready for discharge but admitted that they were unprepared to cope with fatigue, decision-making, diet management, reduced fitness, the presence of latent anxiety and existential thoughts.

Humans can imagine future consequences; this was a source of motivation clearly prominent in the participants’ own preparation for allo-HCT. Motivation is primarily about the activation and persistence of behaviour and is connected to cognitive activities. Expectations are a determining factor in people’s choice of activities; how much effort they will need to expend, and how long they will endure and cope in stressful situations [38]. When living in, and dealing with, a body that is perceived different after treatment, it is important that the patient is invited to dialogue with HCPs to discuss their needs, motivation level and what to expect [39, 40].

According to the participants, the HCP provided ample information, mainly before admission and at discharge. The participants described that they needed time to digest the information given. Gradually informing the patients over time was not applied even though the length of care was approximately 1 month. Mediated information can be described as information the HCPs (experts) consider important for the patient (recipient of care) and therefore design from a biomedical perspective [41]. This can preserve the view of the patient as a passive receiver of care instead of a co-creative contributor [42, 43]. Risks associated with treatment need to be communicated, but it was clear that the participants had individual preferences regarding information where the use of medical terms and the timing of the information may impact patient understanding [44].

In this study, the participants’ own preparations proved to be responsible and extensive, and their self-efficacy to manage self-care was good. Self-efficacy was good before admission when patients sought information to understand the transplant process by contacting patient organizations, social media or searching for information on the internet. However, this valuable information about the participants’ resources and abilities was neither shared nor requested by the RNs or other HCPs. The patient narrative is an opportunity for rewarding encounters between RNs, HCPs and patients, contributing to educational interventions [1, 45]. Person-centred care has a forward-looking stance, facilitating patients to be active in their care and find solutions in partnership [46]. By using open-ended questions, a more person-centred dialogue can be encouraged followed by attentive listening [47]. Two of the 14 identified basic care needs in nursing are care for communication and education [48], so RNs in allo-HCT can strengthen their professional role through prioritising planning, communication and education in their meeting with the patient [49, 50].

## Methodological considerations

A convenience sampling strategy was applied to include participants with experience of the subject, but with a spread regarding age and gender. However, the majority of the participants were women. This may affect the results since gender differences in allo-HCT include poorer emotional well-being, more fatigue and impaired quality of sleep among women compared to men [51]. Among the participants, the age groups 40, 50, 60 and 70 years are represented, but no younger adults are included, which is a limitation. Furthermore, recruitment via self-selection can lead to participants who sign up having strong opinions, expressing dissatisfaction or having a different agenda than the purpose of the study. This was not something that was noticed among the participants in the study. A low number of participants may be perceived as a limitation, but information power was used as a guideline for sample size. A narrow aim and rich, informative narratives require a smaller sample size [25]. The interview guide was pre-tested by patient members of the Swedish Blood Cancer Association regarding usability and comprehensibility. During the analysis, a regular dialogue was conducted within the research group about the sorting and interpretation of data [44]. The interviews reflect each individual’s experience and cannot be transferred to group level. They provide valuable knowledge about preparations carried out independently and the experience of preparations given by HCPs in the allo-HCT process. This knowledge can be primarily directed at HCPs and applied in future care encounters with patients.

## Conclusion

Both participants and HCPs prioritised preparing for allo-HCT in the period before admission. During the period of care, the preparation decreased as the time was not used for preparatory learning. This approach suggests that participants were prepared for the acute phase but less prepared for life after allo-HCT. Self-efficacy was experienced as high among the participants who sought information about and solutions to taking care of their health and everyday life.

## Data Availability

Raw data are not publicly available to preserve individuals’ privacy under the European General Data Protection Regulation.
